# Does support for the legal right to an abortion differ across generations in the United States?

**DOI:** 10.1371/journal.pone.0341223

**Published:** 2026-03-09

**Authors:** Carlisle Rainey, Robert A. Jackson

**Affiliations:** Department of Political Science, Florida State University, Tallahassee, Florida, United States of America; National Cheng Kung University, TAIWAN

## Abstract

The Supreme Court’s ruling in *Dobbs v. Jackson Women’s Health Organization* (2022) has reinvigorated interest in public opinion regarding abortion rights. Recent cross-sections of polling data reveal that young adults are strongly pro-choice and markedly more pro-choice than older adults—with accompanying commentary frequently reporting on the especially pro-choice attitudes of Generation Z and the prospect of the Court’s ruling becoming starkly counter-majoritarian. However, analysis of a single cross-section cannot distinguish *differences between generations* from *differences between younger and older respondents*. We examine the generational differences hypothesis carefully. Do data support the claim that attitudes toward the right to an abortion vary across generations? Analyzing five decades of General Social Survey (GSS) data, we use Bayesian hierarchical models to estimate the differences in respondents’ attitudes toward the legal right to an abortion in the United States across ages, survey years, and generations (specifically, the Silent Generation, Baby Boomers, Generation X, Millennials, and Generation Z). We find large differences in attitudes across ages and especially survey years, but only small differences across these generations. Beginning at about the age of 40, Americans have followed a consistent trajectory of increasingly conservative abortion attitudes. Finally, the large differences across survey years indicate that American public opinion toward abortion is malleable in the near term—with the data revealing contemporaneous, directional shifts in attitudes across society.

## 1. Introduction

*Dobbs v. Jackson* [1] overturned *Roe v. Wade* [2] and placed abortion policy under a bright and hot political spotlight once again. Pew Research Center data [3] indicate that a majority of the national public did not approve of the Supreme Court’s decision in *Dobbs* (see also [4]). However, this counter-majoritarian ruling from a set of unelected elites places the legality of abortion back into the hands of elected officials—with the short-term outcome being widely varying state-to-state policy [5]. Now under the control of elected officials, abortion policy may become more responsive to (state) public opinion. The Pew data also reveal that young adults were the least supportive of *Dobbs* (see also [6]) and attendant discussion frequently suggests that *Dobbs* may become increasingly counter-majoritarian (at least nationally) as the members of the current oldest generations (the Silent Generation and Baby Boomers) continue to die off and be replaced in the electorate by Generation Z. A typical headline accompanied Melissa Deckman’s July 2022 opinion piece at *The Hill*: “The Numbers Show Gen Z Is Actually the Pro-Choice Generation” [7]. Several weeks prior Daniel Cox at *FiveThirtyEight* had written that “…we’re left to solve another riddle: Why do Generation Z adults (born between 1997 and 2004) not share millennials’ more conservative perspectives on abortion?”—outlining possible answers in the remainder of his feature [8].

Unfortunately, a major limitation plagues (almost all) reports that claim generational differences in abortion attitudes (and projections that the *Dobbs* ruling inexorably will become increasingly counter-majoritarian): they do not address the long-recognized age-period-cohort (APC) identification problem [9,10]. The widely-adopted generation classifications—Silent Generation, Baby Boomers, Generation X, Millennials, and Generation Z—that have appeared in innumerable polling reports, news accounts, and academic studies actually represent cohorts, albeit lengthy ones. Cohort analyses, implicitly or explicitly, involve three interrelated variables: age, period, and cohort. Each of these variables is a linear function of the other two, such that knowledge of the values of two provides the value of the third. For example, knowing that someone was 20 years old (age) when answering a survey in 2020 (period) provides the respondent’s cohort as based on her birth year of 2000—e.g., a Gen Z designation in the most common generations’ framework. Munger provides a restatement of the longstanding inferential dilemma in his recent treatment of political differences across generations in the contemporary United States [11]:

In a given time period, the distinct effects of age and cohort are impossible to tease apart…. It would be more satisfying if I could analytically separate these two types of causes and estimate the relative magnitude of their effects… (p. 9)

Even scholars at the Pew Research Center, arguably the most visible producer across recent decades of claims regarding generational differences in American public opinion, have recently acknowledged that Pew’s cross-sectional analyses have been incapable of distinguishing between generation effects and age effects and, thus, that the Center’s conclusions regarding generation differences may have been potentially misleading [12]. Unfortunately, a recent report on future practices issued by the Center’s director of social trends research contains the following problematic statement regarding the analysis of changing attitudes on emerging social trends: *All of these stories – rooted in the life cycle, not in generations – are important and compelling, and we can tell them by analyzing our surveys at any given point in time* [13] (italics added for emphasis). Again, a single cross-section *cannot* discriminate between a life cycle process (age effect) and a generation or cohort effect. Whereas Pew’s prior assumption that analyzing a cross-section can reveal generation effects was untenable, simply assuming that age effects operate is also untenable.

Adopting the widely-utilized Pew Research Center political generation classifications, we assess empirically whether abortion opinion differs across the Silent Generation, Baby Boomers, Generation X, Millennials, and Generation Z. *Silent Generation* refers to those born in the years 1928 through 1945, *Baby Boomers* to those born in the years 1946 through 1964, *Generation X* to those born in the years 1965 through 1980, *Millennials* to those born in the years 1981 through 1996, and *Generation Z* to those born in the year 1997 and later. We use many cross-sectional data sets from the 50-year period 1972 through 2022 and Bayesian hierarchical models to describe abortion attitudes across ages, survey years, and generations. Contrary to existing conclusions, we find minimal differences across generations (including Generation Z), especially relative to the differences we find across ages and survey years.

## 2. Theoretical context

Assertions of the importance of generations for political attitudes and behavior have a longstanding tradition in social science. Karl Mannheim’s essay [14] provides a theoretical touchstone on which contemporary research in both sociology and political science continues to draw. Analogizing a generation to a social class, Mannheim indicates that the members of each share a common location in a social structure and the historical process—“predisposing them for a characteristic mode” (p. 291) of thought, experience, and behavior. According to his argument, during a given period, major societal events and ideas imprint especially on those in adolescence and young adulthood, laying the foundation for a distinctive generation going forward (see also Ryder 1965) [15]. Building on Mannheim’s [14] and Stoker’s [16,17] (see also [18]) treatments refer to adolescence through early adulthood as the “impressionable years,” which are pivotal to political socialization. The major events and experiences during this period of life shape individuals’ political identities and attitudes—however, these identities and attitudes stabilize by its end and largely endure throughout the remainder of citizens’ lives.

Whereas a generational explanation looks back to the major societal and political events of an adult’s adolescence to explain contemporary attitudes and behavior, a life-course approach to politics emphasizes a maturational perspective—knowing an adult’s age is important to explain someone’s contemporary attitudes and behavior not so much because that enables a researcher to place her within the context of her youth but because doing so tells the researcher about her current life stage. Braungart and Braungart [19] summarize the life-course approach to explaining political attitudes and behavior:

The assumptions of the life-course approach are that as individuals grow older, they undergo certain qualitative changes in physiology, cognitive functioning, emotional patterns, and needs. These biopsychological changes occur over the life span and are considered to be sequential, irreversible, and for the most part universal. The maturational unfolding process occurs as individuals of similar age levels move in a sequential direction toward certain characteristic growth patterns. (p. 208)

Consistent with this argument are investigations that conclude that people have characteristic ways of thinking and behaving at various stages of life—e.g., young people tend to be politically liberal, and the middle-aged tend to be less liberal than young people, but not as conservative as old people. Contemporary studies lay out complementary arguments—including psychological, physiological, social, and economic—regarding a possible aging influence on conservatism generally [20–22].

Finally, a period effect refers to a generalized shift across the members of a population during a short period of time, regardless of their age or generation. In terms of political attitudes, a potential catalyst could be a societal crisis (e.g., economic, health, or foreign), the passage of a major law or series of laws, or a high-profile Supreme Court ruling, among other things.

## 3. Research on generational and cohort differences in abortion attitudes

Several academic studies have examined generational or cohort differences in abortion attitudes and/or the relationship between age and abortion attitudes. Few directly acknowledge, and precious few have attempted to address empirically, the entirety of the APC identification problem. Rouse and Ross [23] administered a national survey in 2015 to provide data for their book *The Politics of Millennials*. They report that “generational attitudes or experiences” did not drive abortion attitudes—their “distribution…is very similar between Millennials and older adults” (p. 180; see also [24]). However, their cross-sectional data do not enable them to distinguish the potential influence of age from that of generation nor to investigate the possibility of period effects.

Also conducting a purely cross-sectional analysis, Schnittker, Freese, and Powell [25] were acutely aware of the potential confounding of age, period, and cohort effects. Relying on the 1996 GSS, they examine influences on feminist self-identification, a major component of which are abortion attitudes, and conclude that “strong differences across cohorts” exist (p. 607). However, they acknowledge the following:

…we recognize that age and cohort cannot be distinguished in cross-sectional data, and analysts must rely on side information from theory or other data sources in trying to make sense of these effects (Converse 1976) [26]. We *believe* that the literature provides more reason to think of differences among these groups as cohort effects rather than as age effects, *although we urge further research on this point* (italics added for emphasis).

Incorporating recent data from both the GSS and the New Zealand Attitudes and Values Study, Osborne et al. [27] conclude that younger cohorts support abortion rights more than older cohorts, but their cross-sectional data do not enable a differentiation between differences across cohorts and differences across ages.

In a three-decade-old analysis, Cook, Jelen, and Wilcox [28] (see also [29], pp. 58–59) present a cross-sectional dominant analysis that relied primarily on pooled data from the 1985–1988 GSS and conclude that their youngest white cohort, whose members came of age during and after the Reagan presidency, were less supportive of legal abortion than “those who came of age during the formative years of the women’s movement” (the 1960s and 1970s) (p. 33). Again, their analysis cannot distinguish differences across cohorts from differences across ages (nor does it assess differences across periods).

Misra and Panigrahi [30] report evidence of both generational shifts and intra-cohort change (i.e., an aging effect) in abortion attitudes based on an examination of seven cross-sections of GSS data that cover the time frame 1977–1993. However, their analysis cannot distinguish between differences across ages and differences across periods. Relying on National Opinion Research Center (NORC) and GSS cross-sections that cover a more expansive time frame (1965–1994), Scott [31] concludes that intra-cohort shifts are due to period factors rather than aging effects—she arrives at this conclusion by invoking an *assumption* that attitudes, across the board, become more conservative with age. An approach that statistically discriminates between period effects and aging effects would be preferable.

In a two-decades-old review essay, Jelen and Wilcox [32] discuss persistent generational differences in support for legal abortion, indicating that those who came of age before the 1960s were markedly less supportive of abortion than those who reached adulthood later. They conclude:

Throughout the period of 1972-2000, older, more conservative cohorts have been gradually replaced by younger, more liberal ones, yet the overall mean and median on abortion has remained constant. If the population in 2000 was made up of the same cohort distribution as the population in 1972, support for legal abortion would be far lower. Thus, generational replacement *apparently* masks a longer-term secular decline in support for legal abortion…. the predicted pro-choice change in aggregate abortion opinion has *perhaps* been offset by a strong period effect in a pro-life direction (p. 492; italics added for emphasis).

Barringer, Sumerau, and Gay [33] isolate the young adults (those 18–35 years old) in the 1978–1980 GSS (Baby Boomers), the 1998–2000 GSS (Gen Xers), and the 2016–2018 GSS (Millennials). Subsequently, they pool together each of these young adult samples and specify dichotomous variables for Baby Boomers, Gen Xers, and Millennials, making the claim that doing so allows them to assess generational differences in attitudes toward abortion. Confining their analysis to young adults (from three different time periods), their approach does reduce the prospect of confounding life cycle or aging effects, albeit in a blunt and rather unsatisfying way. Unfortunately, their approach does not enable differentiation between generation effects and period effects.

Incorporating a contemporary, hierarchical modeling approach that accommodates age, period, and cohort effects, Clark [34] investigates the gender gap across multiple attitude domains, including abortion, in an expansive study that relies on GSS data. Clark reports some cohort differences in abortion attitudes among women but chose not to focus on (the possibility of) aging effects in her study. To justify this choice, she cites two authors [35,36] as theorizing that aging has a “relatively minor effect on individual-level opinion change once a study accounts for cohort and major life-cycle processes” ([34], footnote 2 on p. 34). However, her dismissal of the possibility of aging effects strikes us as questionable —and accounting for (all) life-cycle processes via control variables is analogous to the tall order of accounting for (all) potential confounders in a cross-sectional regression framework.

In sum, it may be the case that the conclusions of (some of) these various scholars regarding generational or cohort differences in, period influences on, and life cycle effects regarding abortion attitudes are correct, but their empirical analyses do not provide discriminating evidence. To move forward our understanding of how abortion attitudes vary across ages, survey years, and generations, we need a modeling strategy and data that enable differentiation among age, period, and cohort effects. Over the last two decades, we have seen major advances in Bayesian computation and now have collections of survey data that cover a much longer time period. We draw on these advances in our analyses below.

We should note that several of the researchers cited above specify cohorts that do not conform directly to Pew’s popularized generation classifications in their statistical models—some opting for shorter windows and some for alternative cut-points [25,28–30,34,31]. We focus on the Pew classifications because of their prevalence in contemporary discussions (both academic and popular) of political generations and attitudes in the United States, including those focused on abortion. We do not claim that the Pew classifications necessarily have a stronger theoretical grounding than alternative cohort choices (see [37]).

We should also acknowledge and clarify briefly what our analyses assess and, as importantly, what they do not accomplish. Detecting a period effect does not causally identify what event(s) produced an aggregate shift in abortion attitudes across society—although the occurrence of a major event (e.g., the Supreme Court’s issuance of a precedent-setting ruling) could be highly suggestive. Detecting the presence of an age effect does not determine the underlying variables producing change in support for the legal right to an abortion as people age—this effect simply confirms a process operating across the life cycle (see [17]). Finally, detecting a cohort effect does not identify what aspects of a generation’s political socialization and maturation produced its distinctive abortion attitudes. However, our approach does allow us to describe the differences across ages, survey years, and generations in a comprehensive manner, which should motivate and guide subsequent work that attempts to identify and assess causal factors.

## 4. Data and variables overview

We rely on the General Social Surveys (GSS) 1972–2022 Cross-Sectional Cumulative Data File [38]. Specifically, we pool 33 GSS cross-sections that cover the years 1972 through 2022. Throughout our analysis, we use post-stratification weights for the cross sections for which they exist (1988–2022) and the traditional adults-in-household design adjustment for earlier cross sections (1972–1987). GSS respondents for any given cross-section are 18 years of age and older.

Our outcome or dependent variable is *pro-choice attitudes*. To measure *pro-choice attitudes*, we create an additive index based on variables that reflect respondents’ responses to whether or not it should be possible for a pregnant woman to obtain a legal abortion if: 1) there is a strong chance of serious defect in the baby, 2) she is married and does not want any more children, 3) the woman’s own health is seriously endangered by the pregnancy, 4) the family has a very low income and cannot afford any more children, 5) she became pregnant as a result of rape, and 6) she is not married and does not want to marry the man. Thus, *pro-choice attitudes* ranges from zero (the respondents thought abortion should be possible in none of the six scenarios) to six (the respondent thought abortion should be possible in all six scenarios).

We considered two alternative analyses, neither of which changes the substantive takeaways of the study (see the Appendix). First, we modeled the six items separately. While the overall support for each policy varies considerably across questions (e.g., support is much lower when the woman does not want any more children compared to when the woman’s health is seriously in danger), the patterns across ages, survey years, and generations are highly similar to that for the simple additive index. Second, we modeled additive indices for the traumatic circumstances and the elective circumstances separately. Again, this more complex modeling strategy produces conclusions similar to those for the additive index of all six items. The Appendix also provides results by race and gender. The results for white men and white women are substantively similar to the overall results we summarize here—the generational component of the model seems to be the least important and any generational differences tend to be small. The results for black men and black women are less clear; the number of respondents in these groups is too small to draw strong conclusions.

## 5. Modeling strategy

To disentangle the differences across ages, survey years, and generations, we adopt the hierarchical modeling approach discussed in Bell and Jones [39] (see also [40]; see [41] for a detailed explanation of the modeling strategy applied to economic attitudes). Intuitively, we use a simple regression model to estimate the differences across ages for each generation within each survey year. We then use a hierarchical model to link these regressions into a single model. We begin with the usual normal-linear regression ${\text{pro}-\text{choice attitudes}}_{i[g,t]}=N\left(\mu_{i[g,t]},\sigma_{y}^{\text{2}}\right)$, where i indexes the respondent and [g,t] indexes the generation and survey year, respectively. We might read the notation i[g,t], as “respondent i, who is from generation g and was surveyed in year t.”

We model the expected or predicted pro-choice attitudes $\mu_{i[g,t]}$ for an individual respondent as a quadratic function of age, where each of the three regression coefficients varies across the generations g and survey years t $\mu_{i[g,t]}=\beta_{g,t}^{\text{cons}}+\beta_{g,t}^{\text{age}}+\beta_{g,t}^{{\text{age}}^{\text{2}}}$. The quadratic age function accommodates a nonlinear effect. We decompose each of the varying parameters $\beta_{g,t}^{\dot{\}}\backslash )}$ into a component α that varies across generations and a separate component γ that varies across survey years


$$\mu_{i[g,t]}=\overset{\beta_{g,t}^{\text{cons}}}{\overbrace{\left(\alpha_{g}^{\text{cons}}+\gamma_{t}^{\text{cons}}\right)}}+\overset{\beta_{g,t}^{\text{age}}}{\overbrace{\left(\alpha_{g}^{\text{age}}+\gamma_{t}^{\text{age}}\right)}}{\text{age}}_{i}+\overset{\beta_{g,t}^{{\text{age}}^{\text{2}}}}{\overbrace{\left(\alpha_{g}^{{\text{age}}^{\text{2}}}+\gamma_{t}^{{\text{age}}^{\text{2}}}\right)}}{\text{age}}_{i}^{\text{2}}.$$

This setup has three important parts. First, age can have a nonlinear effect on predicted pro-choice attitudes. Second, this pattern can vary across survey years. For any given survey year, the model allows predicted pro-choice attitudes to differ from those for other years. It allows a mean shift, where the predicted attitudes are more or less pro-choice overall. But the model also allows the relationship between predicted attitudes and age to vary across survey years. For example, the predicted attitudes might idiosyncratically shift more in a particular year for younger respondents. Thirdly, and most critically for our purpose, the model allows the relationship between age and predicted attitudes to vary *within each generation*. Again, the model allows both a mean shift within a generation and the relationship between age and predicted attitudes to vary. This is the core claim of the generational differences hypothesis: respondents’ *generation* brings useful information not captured by their age or the survey year. Some popular accounts suggest that generation is *the* relevant feature (not age or survey year) to describe attitudes in United States politics. This model, then, allows us to assess the relative usefulness of generations to describe attitudes toward abortion policies in the United States.

We model each parameter vector α and γ as a multivariate normal distribution


$$[\begin{array}{c} \alpha_{g}^{\text{cons}}\\ \alpha_{g}^{\text{age}}\\ \alpha_{g}^{{\text{age}}^{\text{2}}} \end{array}]\sim N\left([\begin{array}{c} \mu_{\alpha^{\text{cons}}}\\ \mu_{\alpha^{\text{age}}}\\ \mu_{\alpha^{{\text{age}}^{\text{2}}}} \end{array}],\Sigma_{\alpha }\right)\text{ and }[\begin{array}{c} \gamma_{t}^{\text{cons}}\\ \gamma_{t}^{\text{age}}\\ \gamma_{t}^{{\text{age}}^{\text{2}}} \end{array}]\sim N\left([\begin{array}{c} \mu_{\gamma^{\text{cons}}}\\ \mu_{\gamma^{\text{age}}}\\ \mu_{\gamma^{{\text{age}}^{\text{2}}}} \end{array}],\Sigma_{\gamma }\right).$$

This allows us to pool information across survey years and generations as warranted by the data. The posterior distributions of the diagonal elements of $\Sigma_{\cdot }$ allow a systematic estimate of the variability of the parameters across survey years and generations. We fit the model with HMC using Stan [42,43]. Throughout the paper, we report posterior means and 90% equal-tailed credible intervals [44,45].

This approach is well-suited for our purposes for three reasons. (1) The model represents the “generational differences” hypotheses by fitting many separate regressions of abortion attitudes on age within each generation and survey year while (2) *partially* pooling information about the many regressions across generations and survey years. This allows us to assume that the many regressions are different, *but similar*, with the similarity estimated from the data. Lastly, our modeling strategy (3) allows us to compare the fit of our full model that includes generational differences to that of a simpler model that does not include generational differences.

While this approach has been criticized in some applications (see, for example, [39]), our application uses a definition of “generation” that is many years wide. In the Pew Research Center scheme that we follow, each generational cohort is at least 16 years wide. This allows us to precisely estimate the effect of age within each generation for each survey year. While Bell and Jones’s [39] critiques of the traditional APC model have merit, they are more applicable to applications in which age does not vary much within each period-cohort. We demonstrate the effectiveness of our approach via fake data simulation in the Appendix.

## 6. The relative importance of model components

**Fig 1:**
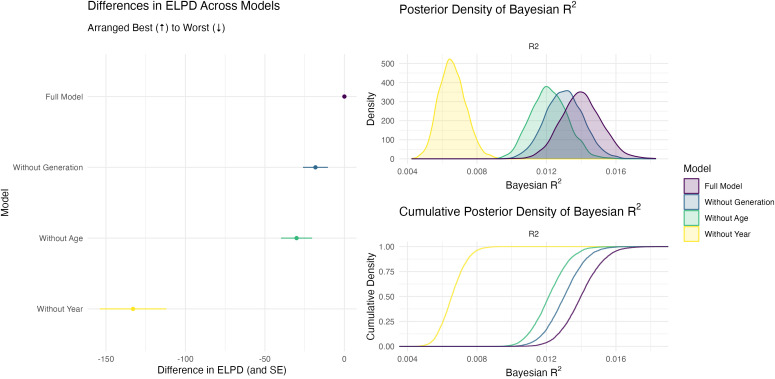
This figure shows the model comparisons between the full model and three models that leave out age, survey year, and generation, respectively. Notice that both the ELPD and the Bayesian R^2^ suggest that generation is the least important of the three components. Generation is much less important than survey year and somewhat less important than age. This suggests that researchers should use caution in interpreting differences between age groups at a single time point as generational differences.

At the most general level, we are interested in the explanatory power of each component of the model: age, survey year (period), and generation (cohort). To provide an initial assessment of the differences across ages, survey years, and generations, we re-fit the model and iteratively omit each component. This gives us four models to compare: the full model, a model without age, a model without survey year, and a model without generation. For each model, we compute the WAIC estimate of the expected log pointwise predictive density (ELPD) and the Bayesian R-squared [46,47]. The ELPD is similar to leave-one-out cross-validation and a measure of out-of-sample predictive accuracy. The Bayesian R-squared is analogous to the usual R-squared in the least-squares framework, except that it is calculated for each posterior sample and therefore has a distribution. Fig 1 shows the differences in the ELPD and the Bayesian R-squared for each model variable.

Fig 1 shows that the full model has the most explanatory power (i.e., the highest ELPD and Bayesian *R*^*2*^), but how much explanatory power is lost when fitting models that exclude the age, survey year, and generation components, respectively? The most obvious change happens when the year component is dropped from the model—without the year component, the Bayesian *R*^*2*^ drops by about 53%. This suggests that, among the three components, the survey year matters most. Notably, *the generation component matters the least*. Excluding the generation component from the model shrinks the Bayesian *R*^*2*^ by only about 7%. Excluding age, on the other hand, decreases the Bayesian *R*^*2*^ by about 13%. In contrast to the popular discourse, generation matters relatively little when compared to age and especially survey year.

This suggests that researchers should exercise caution when comparing attitudes across ages at a single time point (i.e., from a single cross-sectional survey). When referring to differences across age groups as “generational differences,” they are implying that these differences will *persist* because the generational labels will persist. However, it could be that an aging process is in play or that differences are idiosyncratic for that period. This distinction matters, because Generation Z will always be Generation Z, but young people will one day be old. To explore this further, we examine the attitudes across generations, ages, and survey years.

## 7. Attitudes across generations

**Fig 2:**
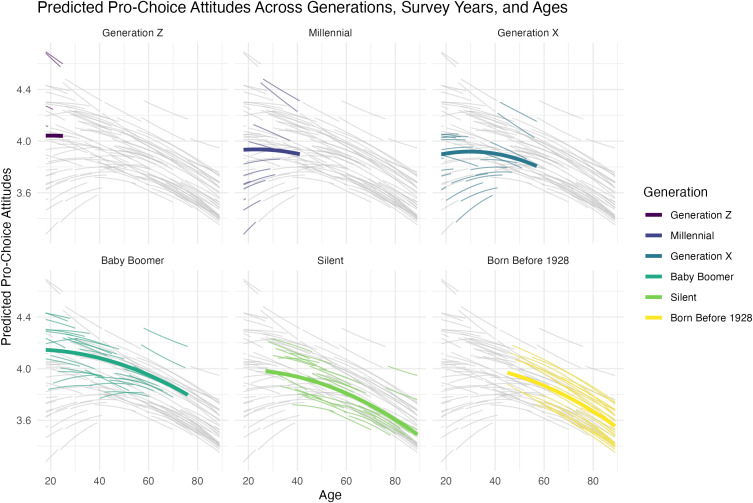
This figure shows the predicted pro-choice attitudes—the number of scenarios (of six) for which respondents agree that abortion should be possible—for each age-generation-year in the data set. The light grey lines in each panel show the predicted attitudes for all age-year-generation groupings. These lines facilitate comparisons across the panels. The fine, colored lines show the predicted attitudes for each survey year. The heavy, colored lines average across survey years to find a typical prediction (across survey years) for each generation. Most importantly, this figure shows relatively little variation in attitudes across generations compared to the variation across survey years and ages.

Fig 2 shows the predicted attitudes from fitting the model into a single display. The outcome variable is the number of scenarios (of six) for which respondents agree that abortion should be legal, so that a prediction of 3.5 means that the respondents within a particular age-generation-year agree that abortion should be legal in 3.5 of the six scenarios, on average. More positive values indicate more pro-choice attitudes. The fine lines show the relationship between age and predicted abortion attitudes for each generation-year. The color of the line indicates the generation. The heavy lines indicate the relationship between age and predicted abortion attitudes for a “typical” year (or marginalizing across survey year differences).

The model suggests substantial variation in attitudes across survey years, but relatively less variation across generations. Further, the variation we do find does not match a narrative that more recent generations have more pro-choice attitudes. Instead, Baby Boomers are the most pro-choice generation. Millennials, Generation X, and the Silent Generation have very similar attitudes—less pro-choice than Baby Boomers. Generation Z falls somewhere in-between, though we have relatively little data for this group so far. And the differences between generations are substantively small, only about 0.1 or 0.2 items of the six, which corresponds to about a 0.05 or 0.1 standard deviation shift in the outcome.

**Fig 3:**
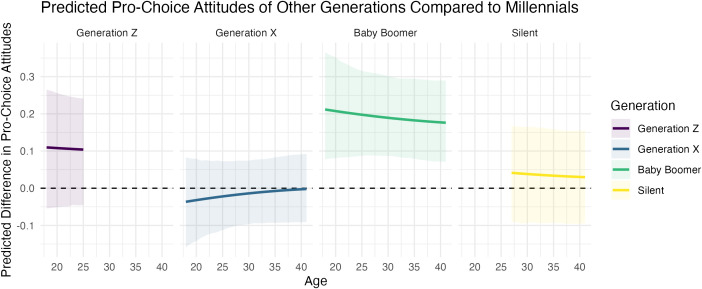
This figure shows the difference between the predicted pro-choice attitudes of other generations and Millennials for each generation-age combination in the data set (averaging across survey years). These are the differences between the heavy line for Millennials in Fig 2 and the lines for the other generations (as well as the 90% credible interval). Most importantly, this figure shows relatively small differences across generations. Generation X and the Silent Generation are very similar to Millennials, and Baby Boomers differ only slightly—the substantive difference is small (about 0.2 items or a 0.1 standard deviation difference on a six-point scale).

Fig 3 explicitly compares the generations using Millennials as the baseline by showing the differences between the heavy lines in Fig 2, as well as the 90% credible intervals. Notice that Generation X and the Silent Generation have attitudes similar to those of Millennials: there is at most a 0.1 items (or a 0.05 standard deviation) difference between these generations. Baby Boomers, on the other hand, hold more pro-choice attitudes than Millennials. However, the difference is still substantively small. There is, at most, a 0.3 item (or a 0.15 standard deviation) difference between Millennials and Baby Boomers.

Finally, we assess the variation in the predicted attitudes for models that marginalize the differences across generations and survey years. First, we use the respondent’s age, generation, and survey year to make the prediction for each case in the data set. We then compute the standard deviation of those predictions, which is 0.23. Then, to assess the relative importance of each component, we marginalize across generations, survey years, and both generations and survey years. “Marginalizing” here can be thought of as generating a prediction for a new survey year, generation, or both. We generate predictions for each marginalization and compute the standard deviation of those predictions for each. Table 1 shows the standard deviation of the predictions for each marginalization.

**Table 1:** This table shows the variation that each component of the model generates in the predicted pro-choice attitudes. Most importantly, respondents’ generation contributes relatively little variation to the predictions.

Marginalization	Source of Variation in Attitudes	Standard Deviation in Predicted Attitudes in the Data Set
None	Age, Generation, and Survey Year	0.23
Generation	Age and Survey Year	0.21
Survey Year	Age and Generation	0.13
Generation and Survey Year	Age	0.11

First, when we marginalize across generations and survey years, the standard deviation in the predictions across the observed ages in the data set is 0.11. This serves as an age-only baseline. When we add the generational differences into the predictions, the standard deviation only increases to 0.13. But when we add survey-year differences into the age-only predictions, the standard deviation jumps to 0.21, about a five times larger jump. Similarly, when we add generations to the predictions using age and survey year, the standard deviation only increases from 0.21 to 0.23. But when we add the survey year to the predictions using age and generation, the standard deviation increases from 0.13 to 0.23. Again, of the age, survey year, and generation components of the model, generation seems the least useful in describing attitudes toward abortion.

## 8. Attitudes across survey years and ages

**Fig 4:**
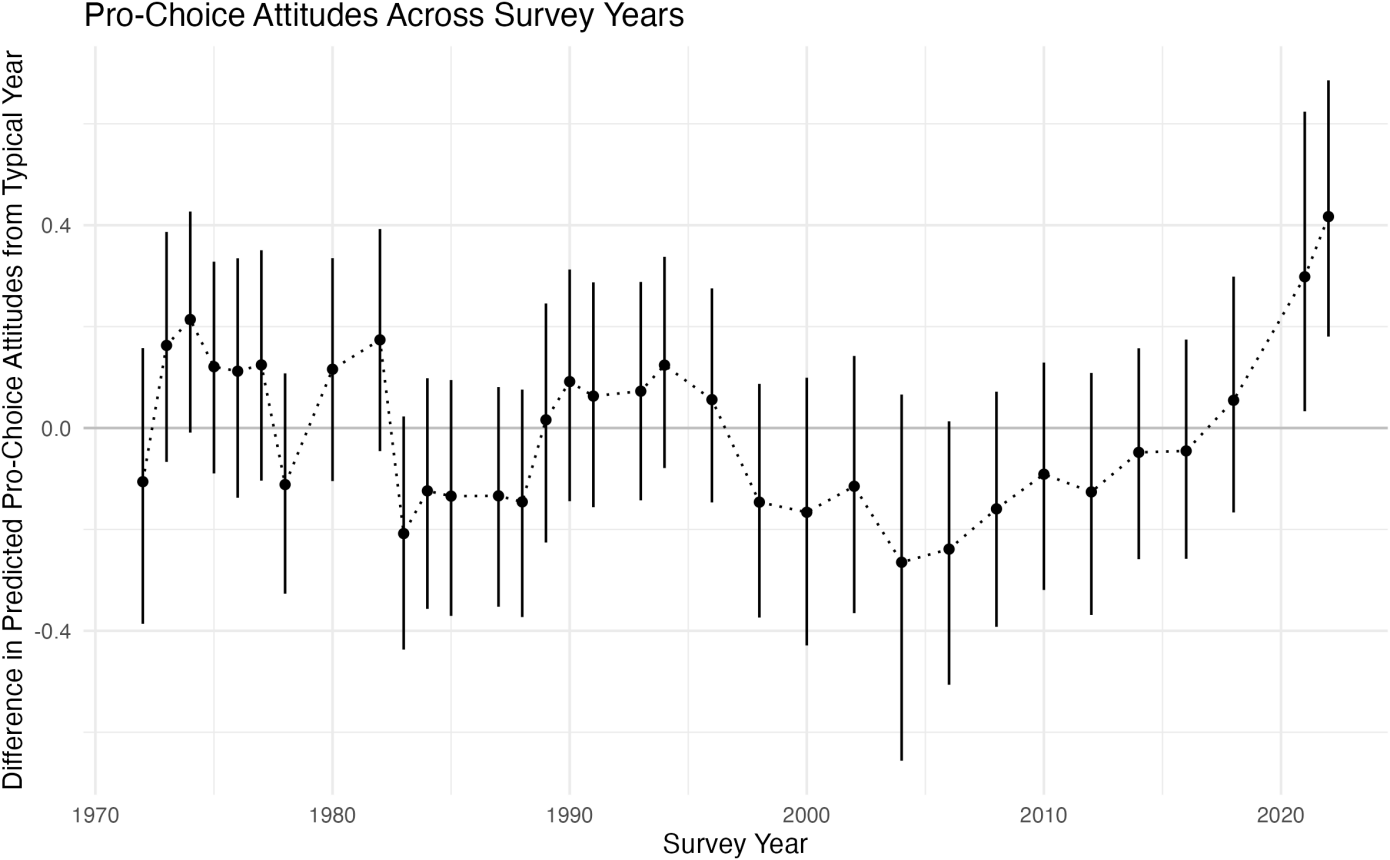
This figure shows the difference between (1) the average predicted pro-choice attitudes across the observed ages in the full data set for each observed survey year and (2) a prediction for a “typical” year that marginalizes the survey year (as well as the 90% credible interval). We find substantially larger differences across survey years than we find across generations.

Whereas we find minimal differences in abortion attitudes across generations, we find larger differences across survey years. Fig 4 shows the differences in attitudes across survey years (compared to a typical year). Specifically, Fig 4 shows the difference between (1) the average predicted attitudes across the observed ages in the full data set for each observed survey year and (2) the average predicted values marginalizing the survey year. Societal attitudes toward abortion shifted in a conservative direction during the Reagan presidency in the 1980s, then reset at a more liberal level across the heart of the 1990s, perhaps the product of a thermostatic shift in response to the rhetoric of the prior decade and the Supreme Court’s rulings in *Webster v. Reproductive Health Services* (1989), *Hodgson v. Minnesota* (1990), *Rust v. Sullivan* (1991), and *Planned Parenthood v. Casey* (1992) [5,48–50]. In the late 1990s and even more so in the early 2000s, society’s attitudes toward legal abortion took a sharp, conservative turn. Since then, they have been drifting in a liberal direction, with the single, most positive pro-choice period effect at the end of our GSS time series in 2022.

The early 2020s witnessed several states enacting laws to restrict abortion, culminating in the Supreme Court striking down *Roe* with its 2022 *Dobbs* ruling. The 2022 GSS had conducted only 17 percent of its interviews before June 24, 2022, the date on which *Dobbs* came down, and more than seven weeks prior on May 2 *Politico* had published a leaked draft of Justice Alito’s majority opinion. Thus, the 2022 period effect likely reflects the influence on near-term opinion of both the leaked draft opinion and the actual ruling (for additional context, see [51–53]).

**Fig 5:**
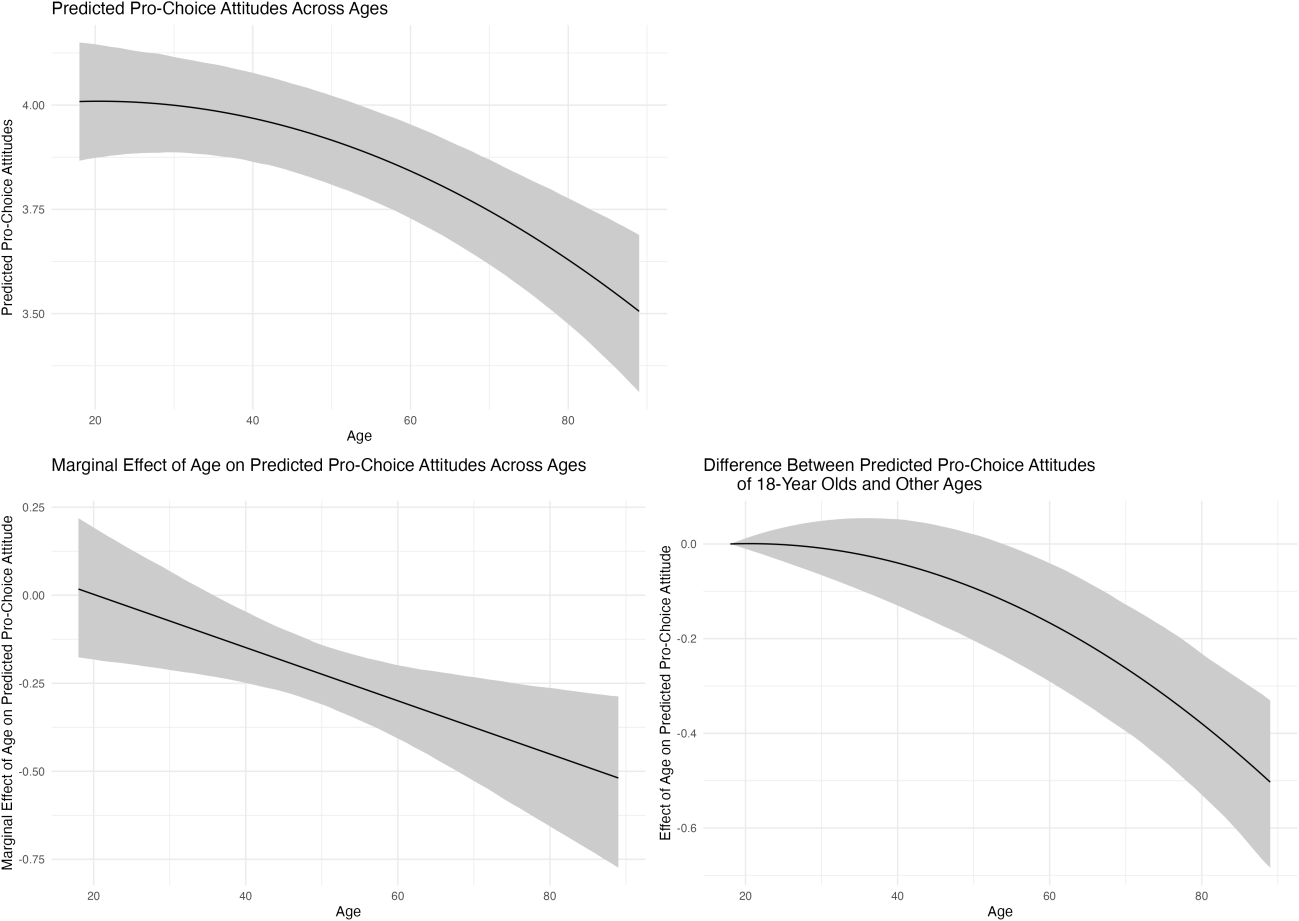
This figure shows the predicted attitudes across ages, the marginal effect of age on predicted attitudes, and the change in the predicted attitudes as age changes from 18 to other ages (as well as the 90% credible interval). In each case, we marginalize across generations and survey years. We find substantially larger differences across ages than generations.

We find substantial differences in abortion attitudes across ages. Fig 5 shows (1) the predicted attitudes across ages and (2) the marginal effect of age on predicted attitudes, both marginalizing across generations and survey years. To our knowledge, our study is the first to demonstrate convincingly that abortion attitudes vary with age. On average, support for abortion rights is rather stable, and perhaps trends in a slightly liberal direction, across the first 20 years of adulthood. However, beginning at about the age of 40, Americans follow a consistent trajectory of increasing conservatism in their abortion attitudes. For example, for 25-year-old respondents, the expected number of pro-choice attitudes is 4.01 (90% CI = [3.88, 4.13]). For 80-year-old respondents, the expected number is only 3.63 (90% CI = [3.48, 3.78]), a difference of −0.38 (90% CI = [−0.53, –0.24]) pro-choice responses. A good deal of empirical research reports that Americans become more conservative across the life course in terms of both their symbolic ideology and economic ideology, consistent with and reinforcing folk wisdom [21,22,54–56]. We observe a similar pattern for abortion attitudes. Whereas existing studies of abortion opinion, which do not account for the APC identification problem, highlight generational differences, our findings reveal that changing opinion toward abortion across the life course deserves more attention than scholars have devoted to it. For example, a recent investigation of panel data from New Zealand indicates that the life event of becoming a parent attenuates support for abortion [57] (see also [58]).

## 9. Conclusion

We do not find meaningful differences in support for the legal right to an abortion across generations in the United States, contrary to popular sentiment, numerous studies, and polling reports. Using a modeling strategy that enables us to estimate differences across ages, survey years, and generations, we find that differences across generations are small, especially relative to the differences across ages and survey years. Again, no prior study has incorporated an analysis approach that enabled its author(s) to uniquely identify age, period, and generation effects. We are skeptical of claims that generational replacement, in and of itself, will move abortion opinion in a liberal direction. Instead, we find larger differences across ages and survey years.

While we rely on the most comprehensive data available to answer our question, the main limitation of our study is statistical power. If we had *even more* data, then we could estimate the differences across generations *even more* precisely. Perhaps then we could clearly see some small differences or clear patterns. Nonetheless, the GSS data that we use allow us to clearly rule out large and even moderate differences across generations.

Follow-up questions are what to make of, and how to proceed with, our finding of minimal differences across generations? A somewhat premature answer would be to conclude that generation or cohort theories are simply uninformative in terms of abortion attitudes. A more appropriate takeaway is that the generation classification scheme that pervades contemporary discussion in the United States does not provide meaningful insight into abortion attitudes. Incorporating conventional cut-points and the common nomenclature (Silent Generation, Baby Boomers, Generation X, Millennials, and Generation Z), we largely sidestepped whether this scheme makes theoretical sense. Cohorts demarcated via theorizing about epochal events pertaining to abortion rights specifically may produce more sizable effects. Also, our modeling approach lends itself to the production of a data-driven cohort definition: researchers can compare the explanatory power of cohorts of varying lengths. But using the popularized classifications and accounting for age and period effects, we find that abortion attitudes do not vary markedly across generations.

Multiple studies have begun to demonstrate empirically that Americans become more conservative across their life course in a variety of areas of political opinion—our findings indicate that abortion attitudes can be added to this list. Although existing studies have advanced a variety of plausible theoretical arguments for the association between aging and conservatism, convincing empirical tests of hypotheses that flow from them are few and far between. Research that investigates “why” the association between aging and decreasing support for the legal right to an abortion would be welcome. Finally, students of public opinion should be able to study year-to-year movements in mass support for abortion rights—i.e., our period effects—*which are purged of age and generation effects*. Existing theories regarding what moves public mood (e.g., the thermostatic model [48–50,59]) should be directly applicable in terms of gaining insight into what accounts for this (isolated) period-to-period variation in abortion attitudes.

## Supporting information

S1 Appendix GSS survey methodology details (citation, question wording, and survey weights), fake data simulation demonstrating that the model recovers known generational patterns, and alternative modeling strategies (elective versus traumatic scenarios, individual survey questions, and results by race and sex). (DOCX)
