# Supplementary material for: Does support for the legal right to an abortion differ across generations in the United States?
Source: PLoS One. 2026 Mar 9;21(3):e0341223. doi: 10.1371/journal.pone.0341223 (PMC12970882; doi:10.1371/journal.pone.0341223)
Supplement: S1 Appendix — (DOCX) [file pone.0341223.s001.docx]

# Appendix: GSS Survey Methodology Details

## Citation

Davern, Michael; Bautista, Rene; Freese, Jeremy; Herd, Pamela; and Morgan, Stephen L.; General Social Survey 1972-2022. [Machine-readable data file]. Principal Investigator, Michael Davern; Co-Principal Investigators, Rene Bautista, Jeremy Freese, Pamela Herd, and Stephen L. Morgan. NORC ed. Chicago, 2023. 1 datafile (Release 1) and 1 codebook (2022 Release 1). <https://gss.norc.org/Documents/codebook/GSS%202022%20Codebook.pdf>

## Methodology

We refer readers to the codebook above for details regarding the survey methodology.

## Question Wording

The codebook linked above for the details of the question wordings. Table 1A provides the variables that we use in our analysis and a brief summary of the question wordings.

Table A1. General Social Survey (GSS) Variables Incorporated into Study

| **Cohort and Age** |  |
| --- | --- |
| COHORT | Birth cohort of respondent (i.e., birth year) |
| AGE | Date of Birth recoded into actual age (in years) |
|  |  |
| **Abortion Battery** |  |
| ***Root question:*** *Please tell me whether or not you think it should be possible for a pregnant woman to obtain a legal abortion. . .* | |
| ABDEFECT | If there is a strong chance of serious defect in the baby? |
| ABNOMORE | If she is married and does not want any more children? |
| ABHLTH | If the woman's own health is seriously endangered by the pregnancy? |
| ABPOOR | If the family has a very low income and cannot afford any more children? |
| ABRAPE | If she becomes pregnant as a result of rape? |
| ABSINGLE | If she is not married and does not want to marry the man? |

To increase the sample size, especially for Generation Z respondents, we included respondents from an experimental grid battery in the online survey. These variables have “G” appended (ABDEFECTG, etc.).

## Survey Weights

We use post-stratification weights (WTSSPS) for the 1988 to 2022 cross sections and the original adults-in-household weights (WTSSALL) for 1972 to 1987. We refer readers to the codebook linked above (pp. 36-37) for the details.

##

# Appendix: Fake Data Simulation and Recovery

In this section, we simulate fake data with known generational patterns and show that our model recovers the differences across generations. We simulate a fake outcome $Y_{null}$ by simulating once from the posterior predictive distribution of our fitted model, but setting all generational random effects to zero. This approach generates a fake data set consistent with our estimated differences across ages and survey years, but with any differences across generations removed.

- **Pattern 1 (No differences):** First, we fit the model to $Y_{null}$. By design, there are no differences across generations in this outcome (except sampling error).

We then re-insert three patterns of differences across generations.

- **Pattern 2 (No differences; strong time trends added):** Second, we create “mock” generational effects with a strong linear time trend. To create these trends, we add a linear trend that subtracts one SD from the first year of the series and adds one SD to the last year in the series. We use this fake data set to show that the model does not detect “generational differences” when the outcomes are simple evolving over time.
- **Pattern 3 (“Generation Z is the pro-choice generation”):** Third, we create a pattern in which Generation Z has slightly more pro-choice attitudes than other generation by adding 0.10 SDs to each $Y_{null}$ for Generation Z respondents in the data set. This gives us the fake outcome $Y_{Gen Z}$. This captures the hypothesis that Generation Z holds more pro-choice abortion attitudes than prior generations.^[[1]](#footnote-2)^
- **Pattern 4 (“Younger generations are more pro-choice than older generations”):** Fourth, we create a pattern in which each subsequent generation holds attitudes that are 0.05 SDs more pro-choice than the prior generation. This captures the hypothesis that younger generations are increasingly pro-choice compared to older generations.

For each pattern, we simulated 50 fake data sets, fit our model to each, and reproduce Figure 3. Figure 6 shows the estimates for each fake data set. The solid lines show the generational differences that we manually added to the data set. This clearly shows that the model can recover the generational differences when they exist and does not recover differences when they do not exist.

For example, if Generation Z is actually “the pro-choice generation” (e.g., Deckman 2022), then our model can recover this pattern. If younger generations are increasingly pro-choice, then our model can recover this pattern.

When there are no differences across generations (i.e., Patterns 1 and 2), the model does not recover differences across the generations (even when there is a strong time trend, as with Pattern 2).


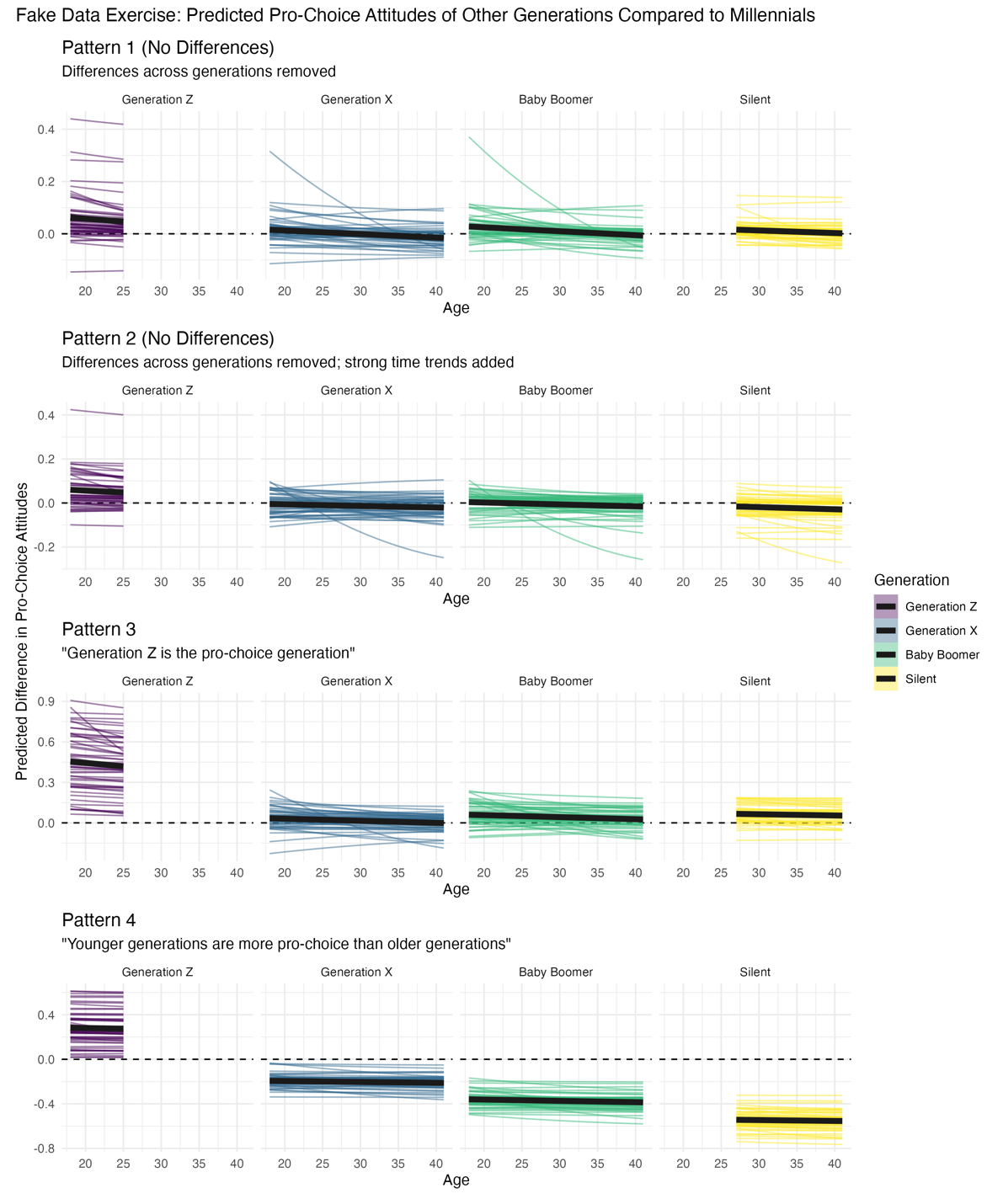


Figure 6: This figure replicates Figure 3 in the main text using three fake data sets in which existing differences across generations are removed and replaced with particular, known differences. In each case the model successfully recovers the correct pattern. This demonstrates that the model can successfully detect differences in pro-choice attitudes across generations when they exist.

# Appendix: Alternative Modeling Strategies

In this section, we re-fit our model on different outcomes and subsets of the data to show that the general conclusion still holds. Recall the general conclusion: although “generational differences” are a common refrain in popular and academic works, we find little evidence that different generations hold different attitudes toward the legal right to an abortion in the United States. We find some evidence of differences across survey years and ages, but once these differences across survey years and ages are held constant, we find little evidence of differences across generations.

1. Breaking the six scenarios into elective scenarios and traumatic scenarios and re-fitting the model on both outcomes.
2. Re-fitting the model on each of the six individual questions.
3. Re-fitting the model using four demographic subgroups: white women, white men, black women, and black men.

## Elective and Traumatic

First, we break our six-item index into two three-item indices.

- The **traumatic index** includes responses for whether or not it should be possible for a pregnant woman to obtain a legal abortion if: 1) there is a strong chance of serious defect in the baby, 2) she became pregnant as a result of rape, 3) the woman’s own health is seriously endangered by the pregnancy.
- The **elective index** includes responses to whether or not it should be possible for a pregnant woman to obtain a legal abortion if: 4) the family has a very low income and cannot afford any more children, 5) she is married and does not want any more children, and 6) she is not married and does not want to marry the man.


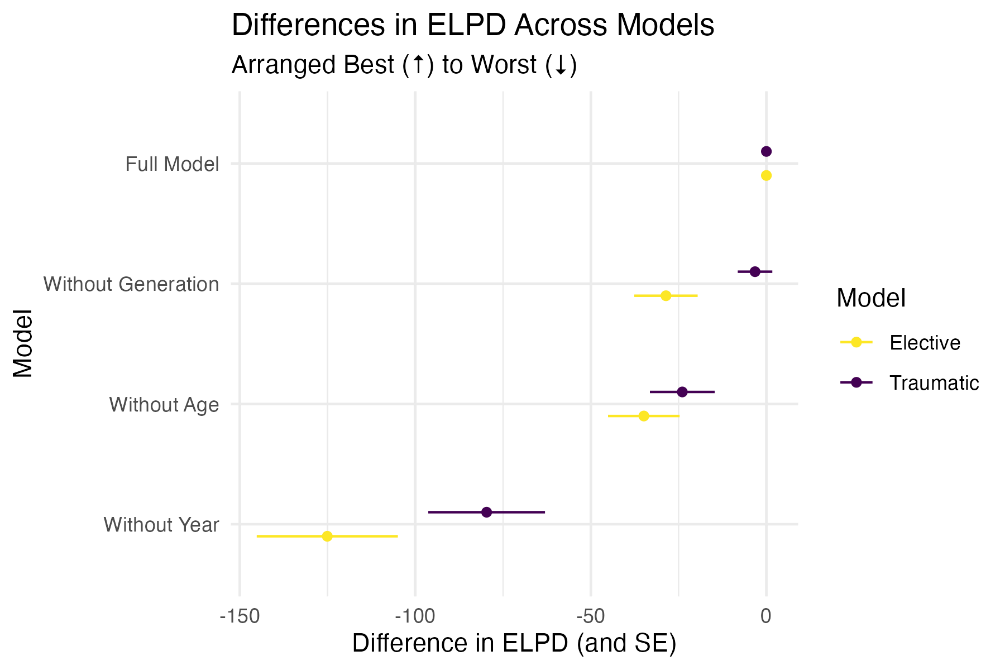


Figure 7: This figure shows the model comparisons between the full model and three models that leave out age, survey year, and generation, respectively, for the elective and traumatic indices. In both cases, notice that the ELPD suggests that generation is the least important of the three components. Compare to Figure 1 in the main text.

##
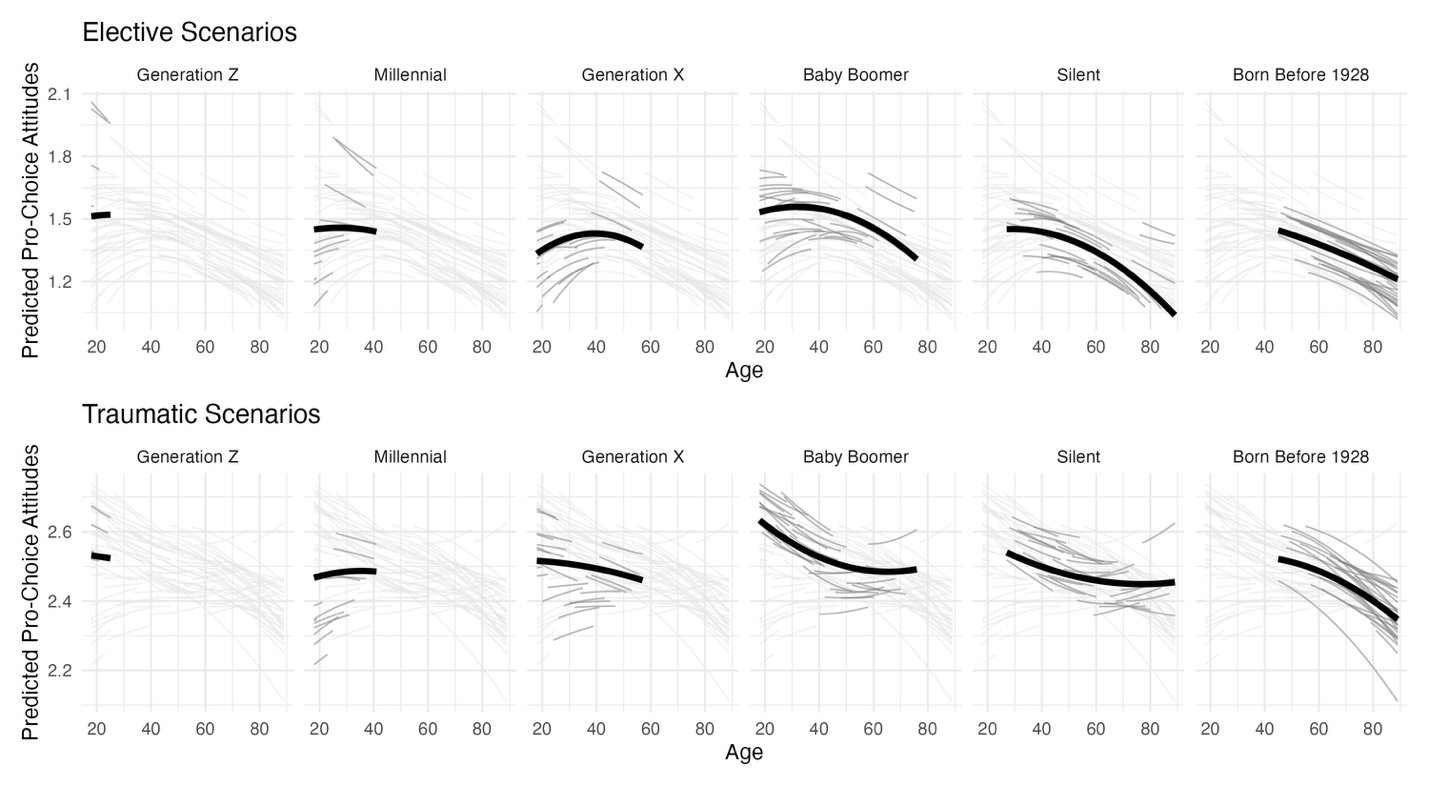


Figure 8: This figure shows the predicted pro-choice attitudes separately for the elective and traumatic indices—the number of scenarios (of three) for which respondents agree that abortion should be possible—for each age-generation-year in the data set. The light grey lines in each panel show the predicted attitudes for all age-year-generation groupings. These lines facilitate comparisons across the panels. The slightly darker lines show the predicted attitudes for each survey year. The heavy, colored lines average across survey years to find a typical prediction (across survey years) for each generation. For both indices, this figure shows relatively little variation in attitudes across generations compared to the variation across survey years and ages. Compare to Figure 2 in the main text.


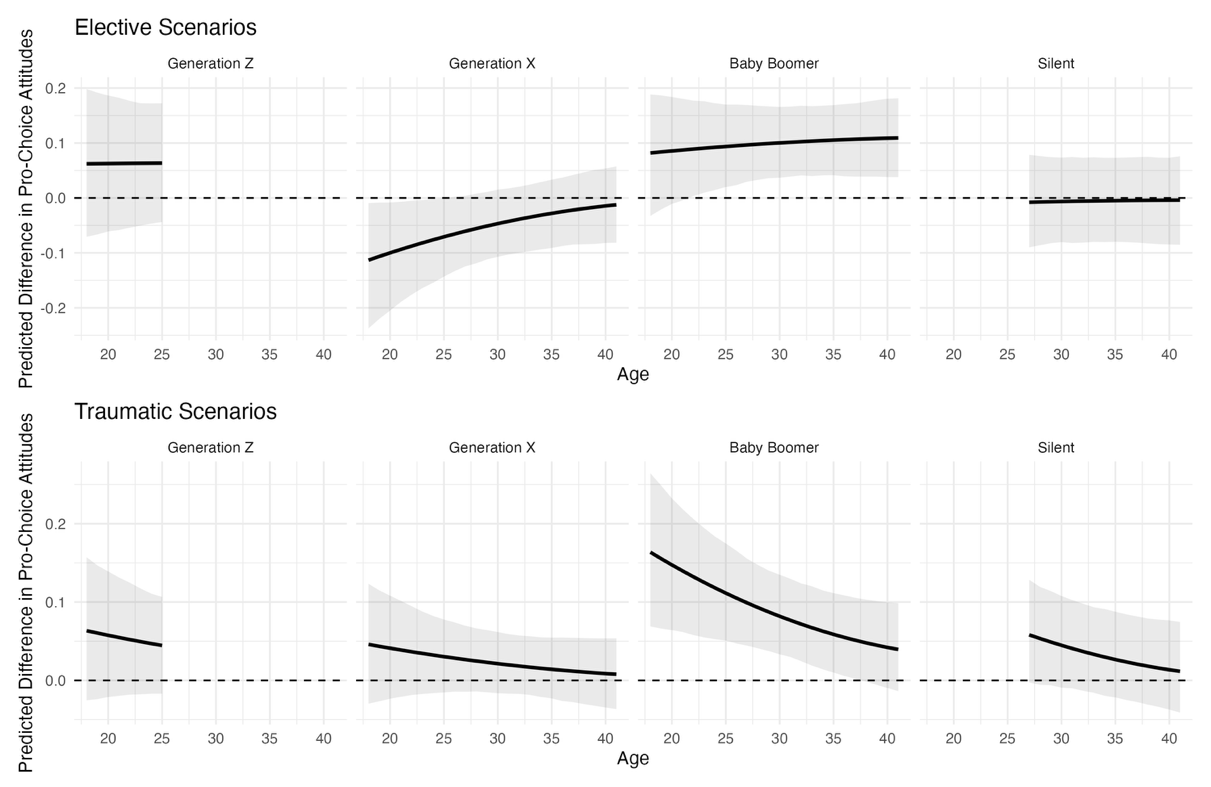


Figure 9: This figure shows the difference between the predicted pro-choice attitudes of other generations and Millennials for the elective and traumatic indices for each generation-age combination in the data set (averaging across survey years). These are the differences between the heavy line for Millennials in the previous figure and the lines for the other generations (as well as the 90% credible interval). Most importantly, this figure shows relatively small differences across generations. Compare to Figure 3 in the main text.

## Individual Questions

Second, we model responses to each of the six items separately. In this case, we use a Bernoulli distribution to model the binary responses rather than a Gaussian distribution.


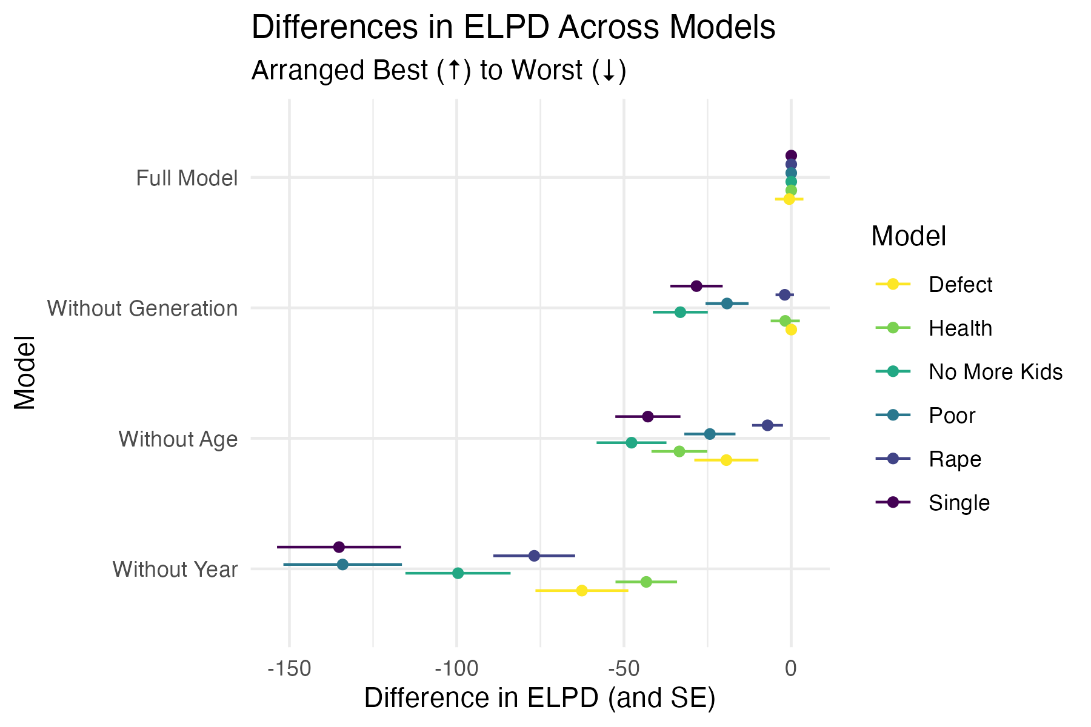


Figure 10: This figure shows the model comparisons between the full model and three models that leave out age, survey year, and generation, respectively, for the each of the six scenarios individually. In each case, notice that the ELPD suggests that generation is the least important of the three components. Compare to Figure 1 in the main text.


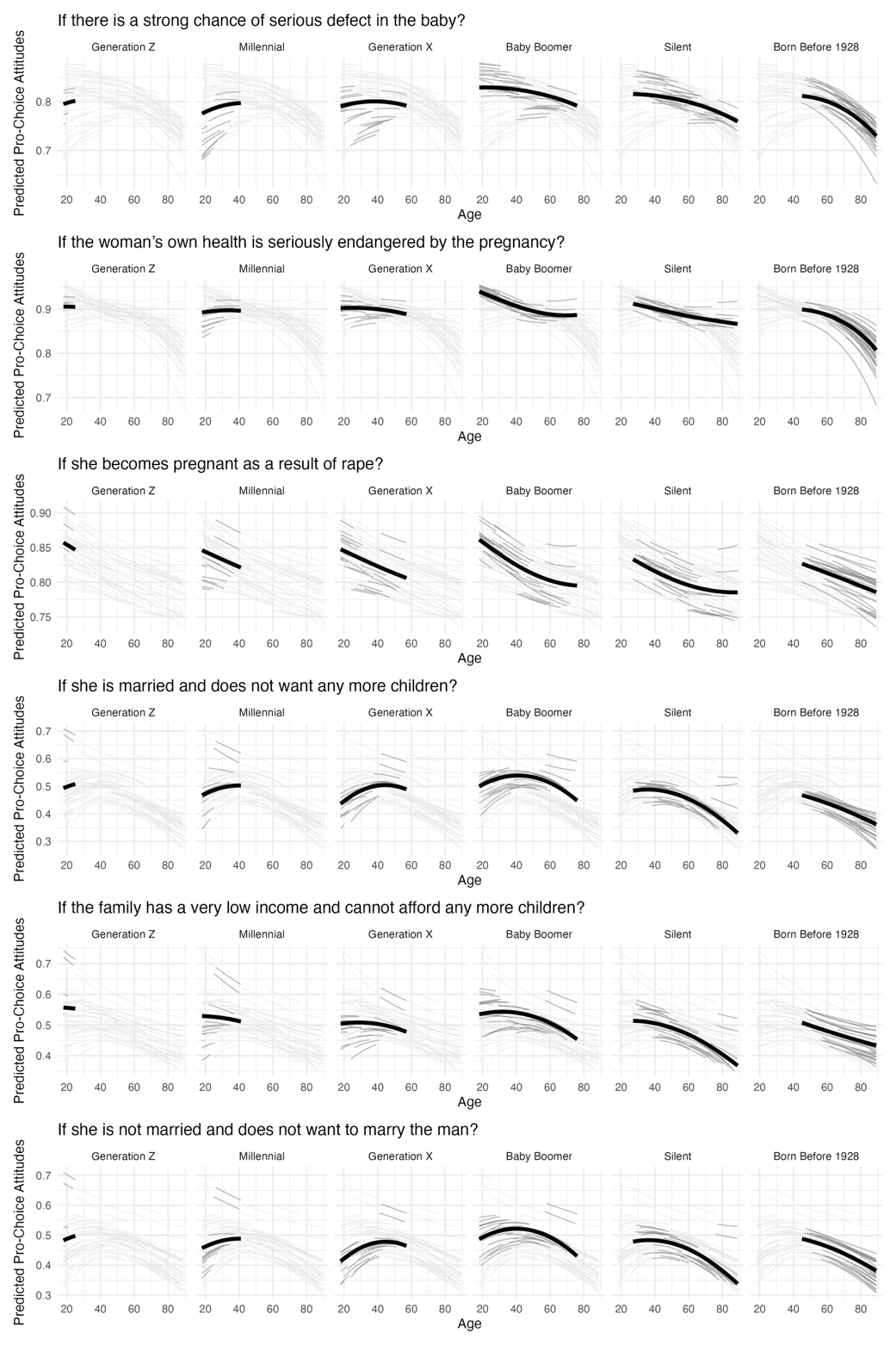


Figure 11: This figure shows the probability of a pro-choice response separately for each individual scenario for each age-generation-year in the data set. The light grey lines in each panel show the predicted attitudes for all age-year-generation groupings. These lines facilitate comparisons across the panels. The slightly darker lines show the predicted attitudes for each survey year. The heavy, colored lines average across survey years to find a typical prediction (across survey years) for each generation. Regardless of the survey question, this figure shows relatively little variation in attitudes across generations compared to the variation across survey years and ages. Compare to Figure 2 in the main text.


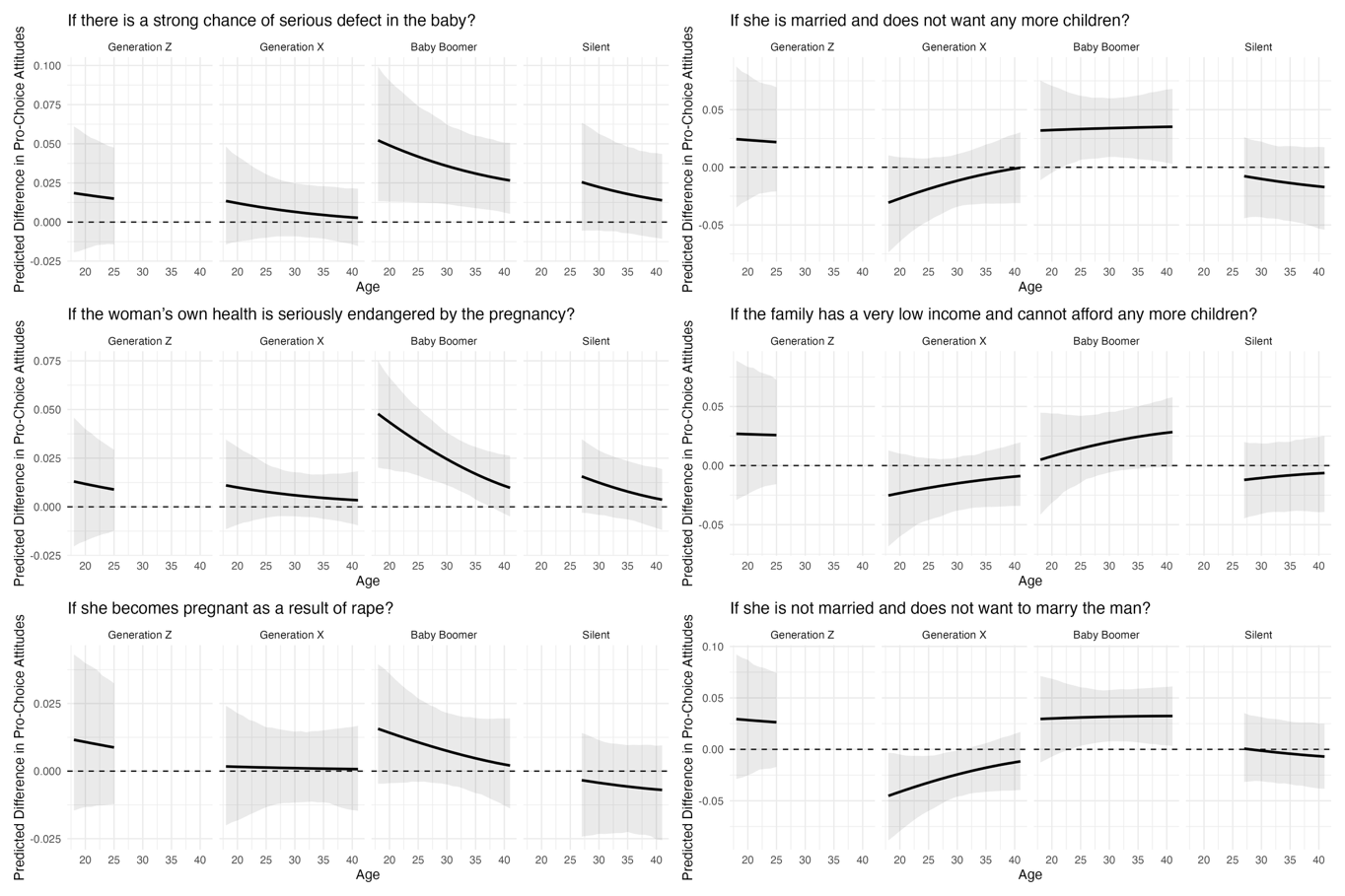


Figure 12: This figure shows the difference between the probability of a pro-choice response of other generations and Millennials for each of the six scenarios individually for each generation-age combination in the data set (averaging across survey years). These are the differences between the heavy line for Millennials in the previous figure and the lines for the other generations (as well as the 90% credible interval). Most importantly, this figure shows relatively small differences across generations. Compare to Figure 3 in the main text.

## By Race and Sex

Third, we model responses to the six-item index separately by race and sex using the coarse GSS categories: white women, white men, black women, and black men.


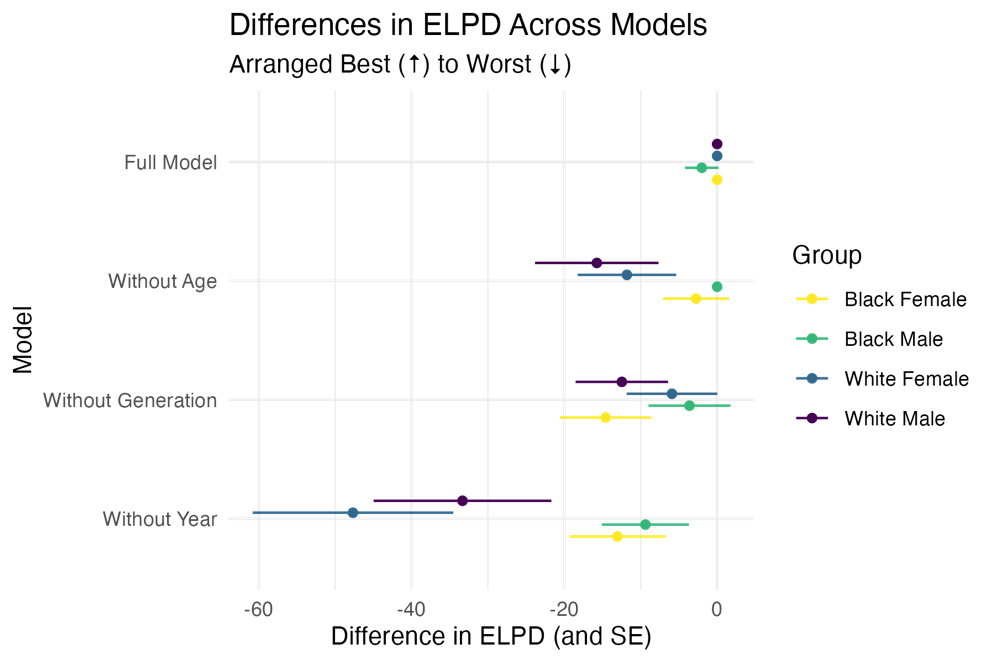


Figure 13: This figure shows the model comparisons between the full model and three models that leave out age, survey year, and generation, respectively, for white women, white men, black women, and black men. Among both white women and white men, notice that the ELPD suggests that generation is the least important of the three components. However, among black women and black men, the pattern is less clear. While perhaps the patterns of attitudes by age, survey year, and generation differ between black respondents and white respondents, it is also the case that we simply have much less data from black respondents. In our data, we have about 160 black respondents per survey and about 1,100 white respondents. Compare to Figure 1 in the main text.


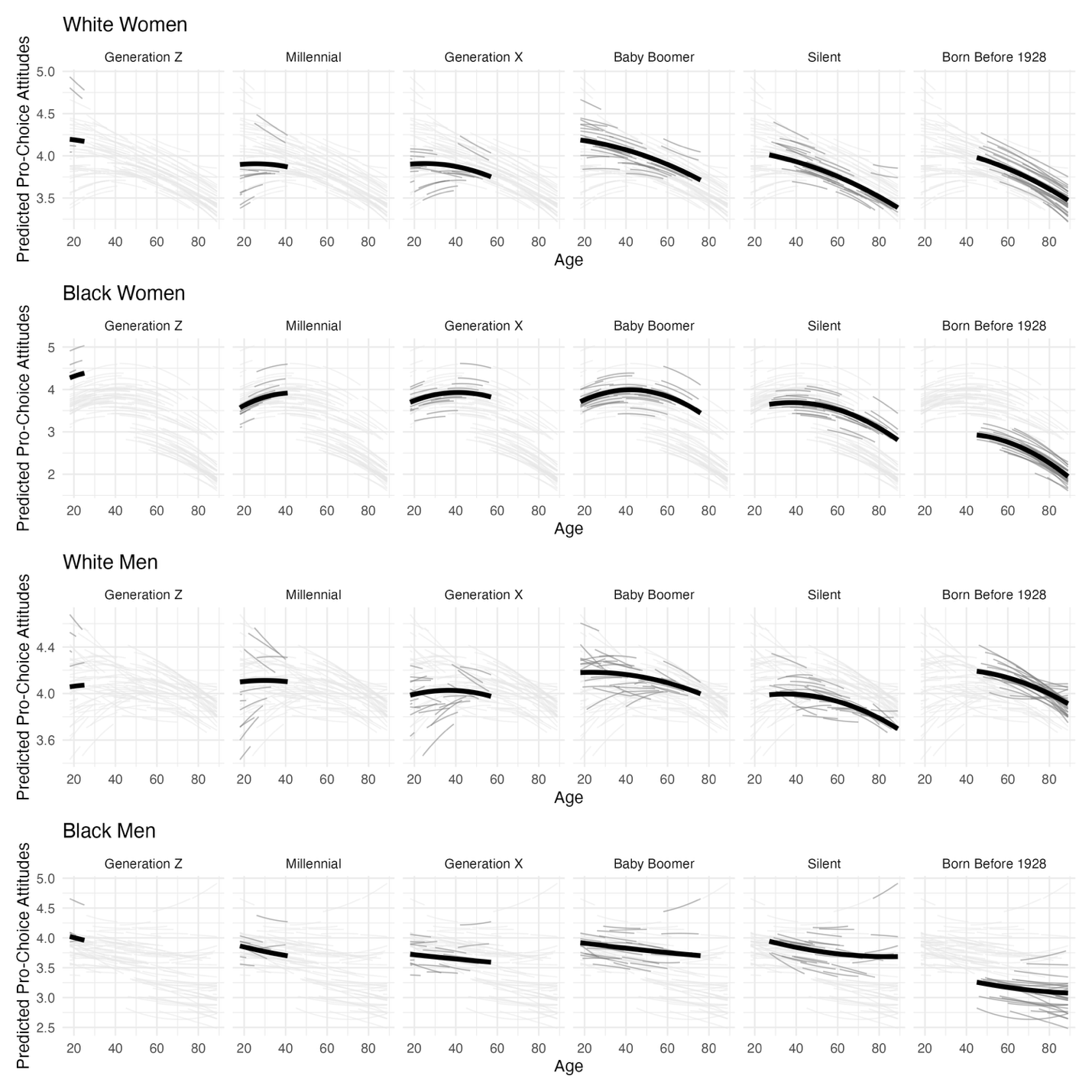


Figure 14: This figure shows the predicted pro-choice attitudes separately for white women, white men, black women, and black men for each age-generation-year in the data set. The light grey lines in each panel show the predicted attitudes for all age-year-generation groupings. These lines facilitate comparisons across the panels. The slightly darker lines show the predicted attitudes for each survey year. The heavy, colored lines average across survey years to find a typical prediction (across survey years) for each generation. Regardless of group, this figure shows relatively little variation in attitudes across generations compared to the variation across survey years and ages. Compare to Figure 2 in the main text.


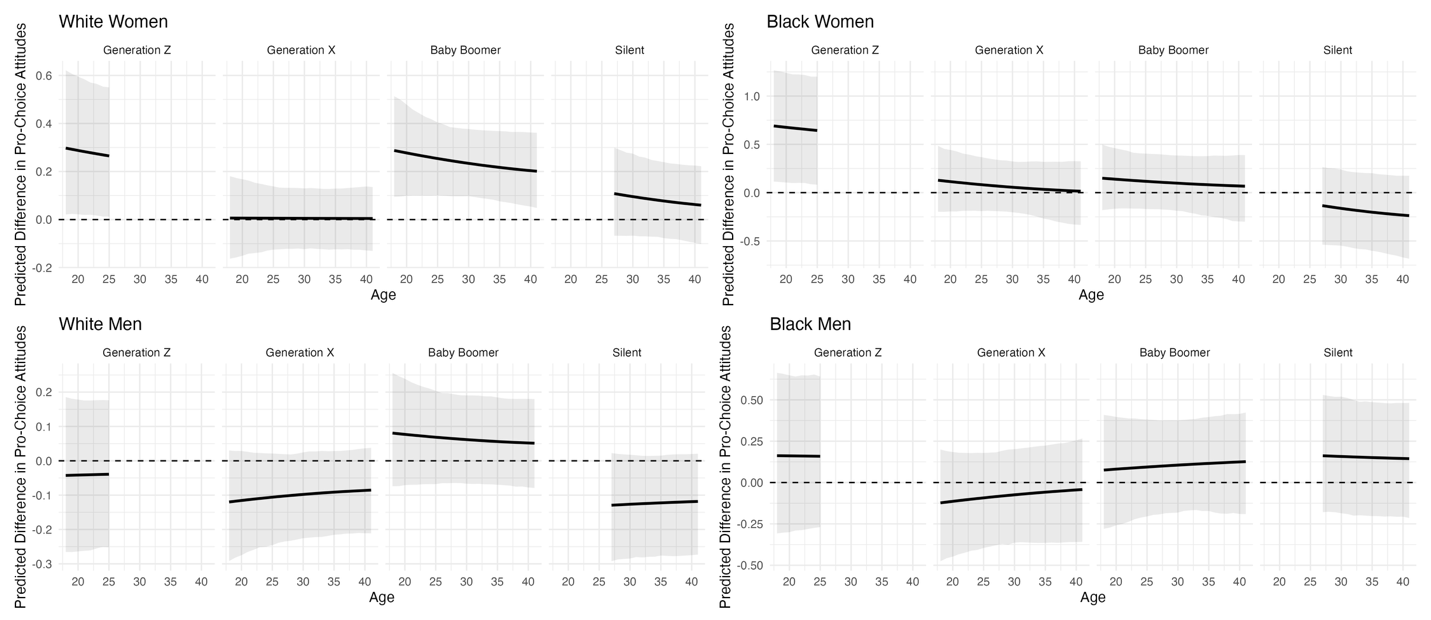


Figure 15: This figure shows the difference between the predicted pro-choice attitudes of other generations and Millennials for white women, white men, black women, and black men for each generation-age combination in the data set (averaging across survey years). These are the differences between the heavy line for Millennials in the previous figure and the lines for the other generations (as well as the 90% credible interval). Most importantly, this figure shows relatively small differences across generations. Among white and black women, the credible interval for Generation Z barely excludes zero perhaps suggesting a difference between Generation Z women and Millennial women. Similarly, among white women, the credible interval for Baby Boomer excludes zero, suggesting a difference between Baby Boomer women and Millennial women. However, overall, we do not find systematic differences in pro-choice attitudes across generations within these groups. Compare to Figure 3 in the main text.

1. Recall these quotations from the introduction to the paper: “The Numbers Show Gen Z Is Actually the Pro-Choice Generation” (Deckman 2022) and “…we’re left to solve another riddle: Why do Generation Z adults (born between 1997 and 2004) not share millennials’ more conservative perspectives on abortion?” (Cox 2022). This third pattern creates a data set consistent with these substantive claims (and our model can recover this pattern). [↑](#footnote-ref-2)
